# Case Report: Mechanical hemolysis resulting from left ventricular outflow tract obstruction after aortic valve replacement relieved by transapical beating-heart septal myectomy

**DOI:** 10.3389/fcvm.2024.1410222

**Published:** 2024-07-11

**Authors:** Qingwen Kang, Jie Tian, Ying Zhu, Wei Zhou, Xiang Wei, Yani Liu

**Affiliations:** ^1^ Department of Medical Ultrasound, Tongji Hospital, Tongji Medical College, Huazhong University of Science and Technology, Wuhan, Hubei, China; ^2^ Department of Cardiovascular Surgery, Tongji Hospital, Tongji Medical College, Huazhong University of Science and Technology, Wuhan, Hubei, China

**Keywords:** left ventricular outflow tract obstruction, hematuria, transapical beating-heart septal myectomy, echocardiographic imaging, case report

## Abstract

**Background:**

Aortic stenosis (AS) in combination with left ventricular outflow tract obstruction (LVOTO) has occasionally been reported. However, making a precise diagnosis and successfully treating this combination is challenging due to the hemodynamic interaction between the two conditions.

**Case summary:**

A 56-year-old male patient who had been diagnosed with severe AS and asymmetric left ventricular hypertrophy underwent aortic valve replacement (AVR) and a conventional septal myectomy. Immediately after the procedure, significant systolic anterior motion and mitral regurgitation developed, necessitating a surgical mitral edge-to-edge repair. Ten days after the procedure, the patient developed hematuria and LVOTO, which was confirmed by echocardiography. Because the LVOTO might have been the cause of the hematuria, the patient underwent alcohol septal ablation, but this had little effect. Three months later, a transapical beating-heart septal myectomy (TA-BSM) was performed in our hospital. Postoperatively, the LVOTO had been significantly ameliorated and the hematuria had resolved.

**Conclusion:**

For patients with AS and LVOTO due to a hypertrophic interventricular septum, inadequate amelioration of the LVOTO after AVR may lead to severe hemolytic hematuria. TA-BSM is a minimally invasive, safe, and effective surgical procedure for ameliorating LVOTO in patients with aortic valve prostheses.

## 1 Case presentation

A 56-year-old man was admitted to our hospital due to left ventricular outflow tract obstruction (LVOTO) and hematuria that developed following an aortic valve replacement (AVR) and septal myectomy (SM) in another hospital. According to the previous hospitalization records of the patient, the initial preoperative transthoracic echocardiography (TTE) revealed severe stenosis of an aortic bicuspid valve [aortic valve (AV); area: 0.73 cm^2^] and a high-pressure gradient (PG) across this AV (peak PG: 139 mmHg; mean PG: 78 mmHg). In addition, both TTE and enhanced computed tomography revealed asymmetrical left ventricular hypertrophy with a maximum septal thickness of 25 mm and posterior wall thickness of 13 mm. The left ventricular end-diastolic diameter was 58 mm, and the left ventricular ejection fraction (LVEF) was 52%. At rest, no accelerated blood flow signal was detected in the left ventricular outflow tract (LVOT), and only minimal mitral regurgitation (MR) was observed. On the basis of these comprehensive preoperative examinations, a replacement of the AV with a prosthetic AV (23# ON-X mechanical aortic valve) and a surgical SM (approximately 15 mm in width, 5 mm in depth, and 20–30 mm in length, toward the apex of the hypertrophied myocardium, and distal to the left coronary cusp and right coronary cusp) were performed on the patient under cardiopulmonary bypass (CPB). Immediately after the procedure, intraoperative transesophageal echocardiography (TEE) revealed systolic anterior motion (SAM) and severe MR, with an LVOT peak PG of 132 mmHg. Therefore, surgical mitral valve (MV) edge-to-edge repair was conducted to ameliorate the SAM and MR. Although the patient was returned to the ward without SAM, with a small amount of MR, an LVOT peak PG of 29 mmHg, and an AV peak PG of 12 mmHg immediately after surgery, he presented with nausea, fatigue, and gross hematuria on the 10th day following surgery. After ruling out unrelated urinary disease, repeated TTE revealed rapid systolic flow in LVOT, such that the prosthetic AV was exposed to a jet with a peak PG of 127 mmHg and severe MR, but there was no evidence of periprosthetic leakage. Considering the LVOTO might be the principal cause of the hematuria, and after medical therapy with β-receptor and calcium channel blockers failed to alleviate his LVOTO, an alcohol septal ablation was performed. Unfortunately, this procedure also did not significantly ameliorate LVOTO or the hematuria.

On admission to our hospital, a physical examination revealed the presence of a systolic murmur at the apical and left sternal margins of the patient, as well as a rapid respiratory rate and substantial breath sounds. A series of laboratory investigations were conducted, which yielded the following results: erythrocyte count 1.81 × 10^12^/L (low), hemoglobin level 6.3 g/dl (low), free hemoglobin concentration >0.04 g/dl (high), haptoglobin concentration <0.006 g/dl (low), total bilirubin concentration 41.9 μmol/L (high), direct bilirubin concentration 7.2 μmol/L, indirect bilirubin concentration 34.7 μmol/L (high), urine occult blood 3+, NT-pro BNP concentration 1.0248 × 10^−6^ g/dl (high), and high-sensitivity cardiac troponin I concentration 1.8945 × 10^−7^ g/dl (high). Furthermore, coagulation and thromboelastography tests showed no abnormalities. These findings indicated the presence of hemolytic anemia.

**Figure 1:**
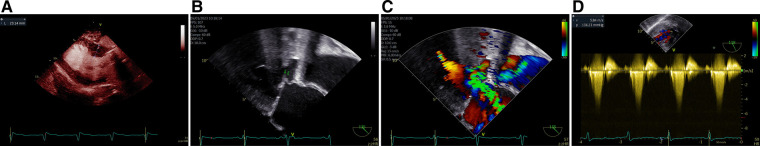
Asymmetric LV hypertrophy identified on the parasternal long-axis view using contrast-enhanced echocardiography (**A**), the anterior leaflet of the MV exhibiting prominent SAM (arrow, **B**), severe MR observed (**C**), and high systolic velocity flow in LVOT reached to 5.8 m/s with a corresponding peak PG of 136 mmHg (**D**).

TTE and TEE were both repeated in our hospital and revealed the following (Figure 1): contrast-enhanced echocardiography confirmed the presence of asymmetrical left ventricular hypertrophy, with the thicknesses of the basal and middle segments of the anterior interventricular septum of 23 and 17 mm, respectively. The thicknesses of the basal and middle segments of the posterior interventricular septum were found to be 16 and 19 mm, respectively. Compared with the initial preoperative echocardiography findings, the left ventricle (LV) end-diastolic internal diameter had decreased from 58 to 45 mm, and the LVEF had increased from 52% to 71%. The LVOT flow was directed into the prosthetic AV with a systolic peak velocity of 5.8 m/s and a peak PG of 136 mmHg. Thus, SAM and an associated MR of 4+ was indicated. TEE showed that the prosthetic AV was clear and opened normally, and there was no perivalvular leakage.

**Figure 2:**
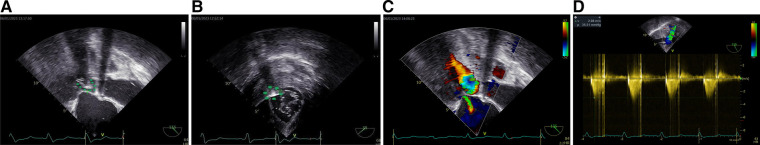
BMD tip visible on the mid-esophageal long-axis view of TEE (arrow, **A**). On the transgastric short-axis view, the BMD was shown close to the anterior interventricular septum (arrow, **B**). After the hypertrophied myocardium was resected, MR reduced to a mild degree (**C**) and the systolic peak velocity in the LVOT decreased to less than 3 m/s with a corresponding peak PG of 35 mmHg, indicating a favorable outcome (**D**).

**Figure 3:**
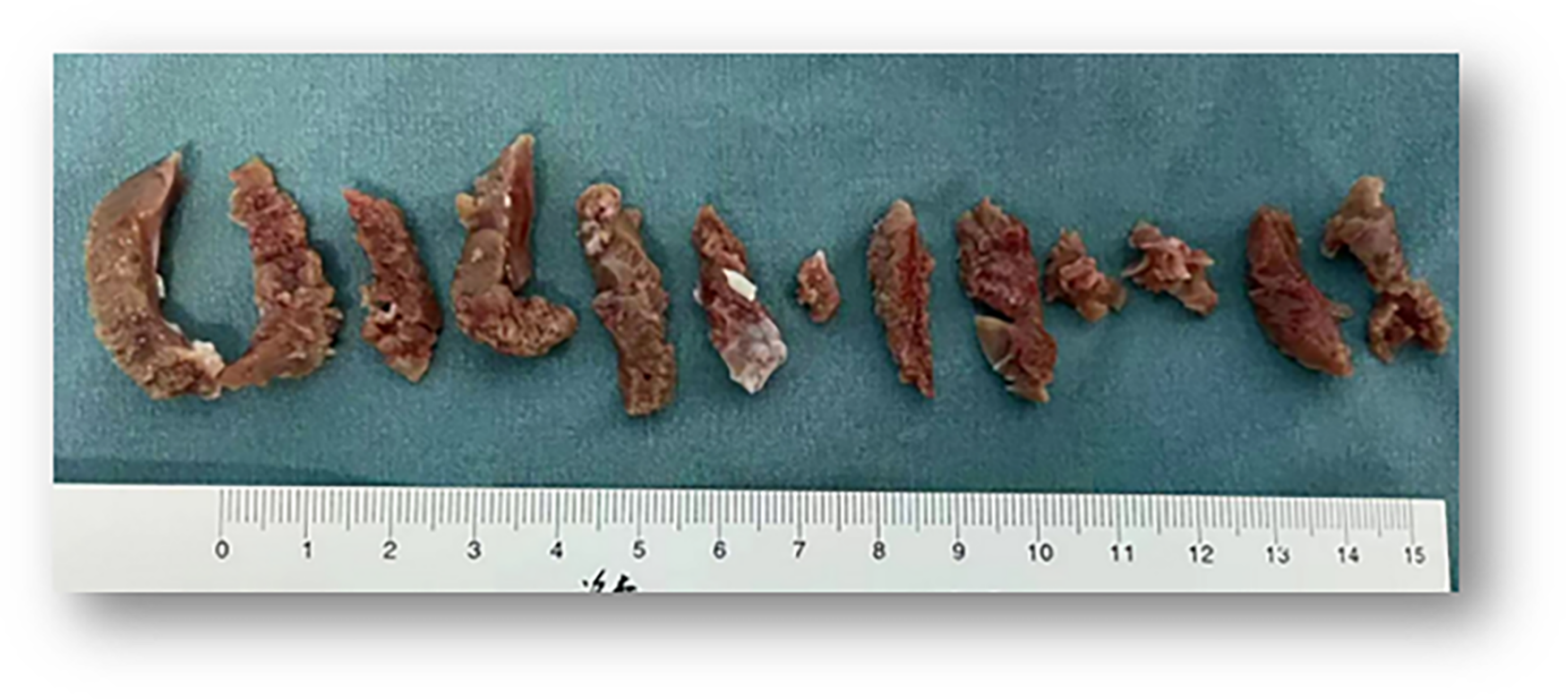
Myocardial tissue following 13 resections.

Because conventional SM through an aortotomy was no longer suitable for this patient, owing to his prosthetic AV, after a comprehensive discussion, a novel minimally invasive procedure, named transapical beating-heart septal myectomy (TA-BSM), was performed (Figure 2). For this procedure, the patient was anesthetized and placed in a supine position. TTE was used to identify the position of the apex within the fifth intercostal space, and then the pericardium was incised and suspended. Subsequently, a double-layered pericardial purse was constructed in the apical avascular area using 3-0 prolene and a felt sheet. Once heparinization had been completed, an arterial manometric catheter was inserted through the purse string to measure the LV pressure. The systolic pressure gradient between the LV cavity and the peripheral artery, which reflects the LVOT gradient, was calculated. The manometric catheter was then withdrawn, and a guidewire was introduced to serve as a guide for the apical dissection. The apex was punctured inside the apical purse string, and then a beating-heart myectomy device (BMD), in the off state, was introduced into the LVOT, guided by TEE. The extent of advancement of the BMD was determined using the mid-esophageal LV long-axis view. The orientation of the resection was identified on the transgastric short-axis view at the basal level (Figure 2). Upon three-dimensional identification of the resection window using TEE, the first resection was performed at the midpoint of the basal anterior septum on the short-axis view, 5 mm away from the AV in the long-axis view. A second resection of the basal anterior septum was performed, parallel but slightly anterior to the first resection, which was achieved by rotating the BMD clockwise from the first resection on the short-axis. Subsequent resections were performed on the basis of a pre-procedure plan and real-time echocardiographic examination until the LVOT peak PG was <30 mmHg or the provoked LVOT gradient was <50 mmHg and the MR grade was ≤1+. A total of 7.8 g of the hypertrophied myocardium after 13 resections was successfully resected (Figure 3). After the incision had been completed, the BMD was extracted, and the apical purse strings were tightened and sutured to stop any bleeding. The entire procedure was performed in the absence of CPB.

**Figure 4:**
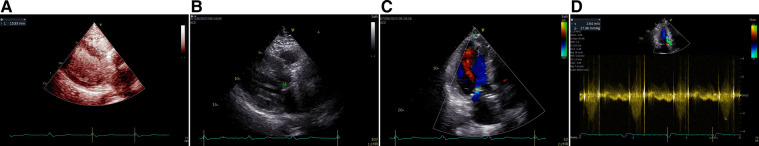
Upon a 7-month follow-up after surgery, the patient's septal thickness was notably thinner using contrast-enhanced echocardiography (**A**). SAM was no longer present (arrow, **B**). Only minimal MR remained (**C**). Systolic peak velocity in the LVOT decreased to 2.6 m/s with a corresponding peak PG of 28 mmHg, indicating a positive surgical outcome (**D**).

The patient was transferred to the ward with an LVOT peak PG of 5 mmHg; his SAM and hematuria had disappeared, even though a small amount of MR persisted. There was no leakage around the aortic valve prosthesis. The patient was pathologically diagnosed as having hypertrophic cardiomyopathy following the procedure but was discharged from the hospital after his hemoglobin concentration had increased and his indirect/direct bilirubin and total bilirubin concentrations had decreased. Seven months later, the patient was found to be in good health, with an LVOT peak PG of 28 mmHg (Figure 4) and no recurrence of the hematuria.

## 2 Discussion

Concomitant hypertrophic cardiomyopathy and aortic stenosis (AS) are common. Typical surgical treatment options include AVR in combination with drug therapy, SM, septal ablation, and mitral repair. Following technological advancements, most patients now experience superior outcomes. Hemolytic anemia caused by worsening LVOTO after AVR has been reported only once, in 2017, and was eventually ameliorated by dual-chamber pacing (1). Here, we report, for the first time, a case of hematuria in a patient with dual obstruction, which developed following AVR and SM and was ultimately alleviated using a novel, minimally invasive TA-BSM.

Accurately diagnosing AS and LVOT obstruction, as well as assessing their severity, presents a challenging task (2, 3). Some researchers have suggested using negative inotropic drugs to alleviate LVOTO before echocardiography to estimate AS (4). In the LV apical five-chamber or three-chamber views, a characteristic “dagger-shaped” envelope on the continuous-wave Doppler can be captured to determine dynamic LVOTO (5). Computed tomography and magnetic resonance imaging can provide precise cardiac structural information. Computed tomography is also a means of quantifying calcification and is increasingly recognized to be the gold-standard method of evaluating the severity of aortic stenosis. However, magnetic resonance imaging is the only non-invasive imaging modality that can be used to assess myocardial fibrosis, and therefore has a distinct advantage for the diagnosis of hypertrophic obstructive cardiomyopathy (3). The present patient underwent cardiac computed tomography, instead of magnetic resonance imaging, prior to the initial surgery. In addition, invasive hemodynamic assessment can be used to preoperatively evaluate the severity of dual obstructions (6). The interaction between AS and LVOTO makes it challenging to accurately assess their respective severities (7). Patients with severe AS have high afterloads, which mask the presence of LVOTO (5). Abnormally rapid intracavitary flow is associated with a small chamber and a hyperdynamic state in patients who have undergone AVR (8–10). In the present patient, the LV end-diastolic internal diameter decreased and LVEF increased after the initial surgery, resulting in the close proximity of the LV walls during systole, thereby reducing local pressure, causing SAM (+) and exacerbating LVOTO. The high-velocity blood flow that impinged on the mechanical aortic valve resulted in the rupture of red blood cells, leading to a high serum hemoglobin concentration and subsequent hemolytic anemia and hematuria.

Beyond the characterization of the double obstruction, the most distinctive aspect of this case study is that, to ameliorate LVOTO, the patient had undergone multiple unsuccessful procedures. Initially, drug therapy is typically used. Aggressive treatment with a β-receptor blocker and a calcium channel blocker is essential to avoid postoperative obstruction, but this was ineffective in the present patient. SM is generally considered to be the preferred treatment option for LVOTO, whether a transaortic, transmitral, or transapical route is used. Despite the risk of septal perforation, the incidence of mortality has decreased significantly with advancements in medical technology. A prospective study, published in 2021, showed that the 1-, 2-, and 5-year survival rates of 191 patients who underwent concomitant AV replacement and SM were 94%, 91%, and 83%, respectively, which are comparable to those of the general US population (11). However, there are some limitations that limit the widespread use of SM. First, stenosis of AV or MV reduces the size of the operative window, making it challenging to determine the extent and range of the septal resection required. Second, it is only after the heart resumes beating that the sufficiency of the myectomy can be assessed. Third, conventional SM is an effective treatment for sigmoid septal hypertrophy, but combined transaortic and transapical approaches are often needed to provide adequate exposure to resolve LVOTO, further increasing the complexity of the procedure and the risk of iatrogenic injury. Therefore, the technology required to perform SM is accessible only to a limited number of large heart research centers. Mitral leaflet edge-to-edge repair is an effective treatment for LVOTO caused by SAM (12). However, in the present patient, the LVOTO could not be solely attributed to SAM because it developed after combined AVR and SM. The obstruction persisted even after mitral leaflet edge-to-edge repair was completed. Owing to its minimal invasiveness, septal ablation of the ventricular septum is more acceptable to patients (13). Prospective studies have shown that the mortality rate of patients within 30 days is 1%, and the survival rates after 1, 5, and 10 years are 98%, 89%, and 77%, respectively (14). However, this procedure is not suitable for patients with a septal thickness <15 mm, no suitable perforating artery of the ventricular septum, or prolonged occlusion of the collateral branches of the target perforating artery (15). There is also a 2% risk of left anterior descending branch stripping, coronary artery spasm, anterior wall infarction, cardiac perforation, and atrioventricular block, which is the most common complication, with 10.5% of patients requiring postoperative implantation of a permanent pacemaker (16).

In response to these limitations, our cardiac specialist team, under the leadership of Dr. Wei, pioneered the development of TA-BSM (17). This innovative approach involves the use of BMD through a mini-thoracotomy, guided by TEE, without the need for CPB. During TA-BSM, real-time TEE provides a comprehensive visualization of LV geometry, which aids the determination of the required extent and range of septal resection. In addition, it permits the measurement of hemodynamic and morphologic parameters after each resection, providing significant advantages over conventional methods. To prevent air and debris embolism, the BMD is flushed with saline to remove any trapped air during the procedure, and the resected myocardium is stored in a negative-pressure chamber until it is removed from the body. The versatility of different BMD models enables the treatment of various subtypes of hypertrophic obstructive cardiomyopathy. Moreover, the absence of CPB during surgery helps to maintain hemodynamic stability.

## Data Availability

The original contributions presented in the study are included in the article/**Supplementary Material**, further inquiries can be directed to the corresponding author.
