# Supplementary material for: Case Report: Mechanical hemolysis resulting from left ventricular outflow tract obstruction after aortic valve replacement relieved by transapical beating-heart septal myectomy
Source: Front Cardiovasc Med. 2024 Jul 11;11:1410222. doi: 10.3389/fcvm.2024.1410222 (PMC11269188; doi:10.3389/fcvm.2024.1410222)
Supplement: Supplementary file 3 [file Datasheet1.docx]

Supplementary Material

## Supplementary Figures

**
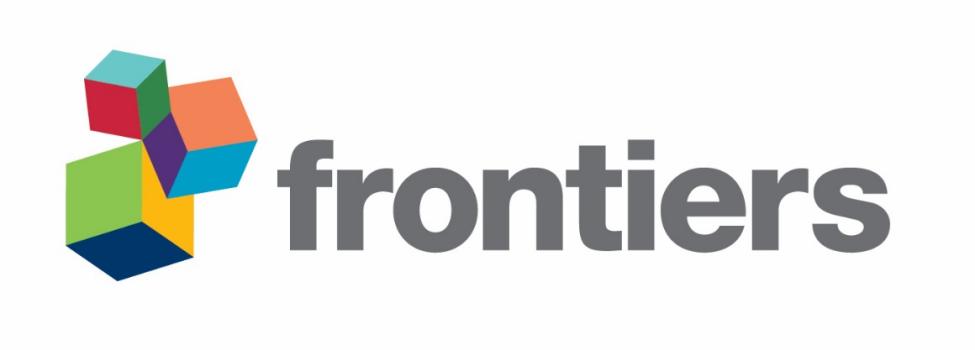
**

**Supplementary Video 1.** The BMD tip is visible on the mid-esophageal long-axis view of TEE (arrow, **A**).

**Supplementary Video 2.** The BMD was then adjusted to approach the anterior septum on the trans-gastric short-axis view (arrow, **B**).

**Supplementary Figure 1A**. Asymmetric LV hypertrophy was identified on the parasternal long-axis view using contrast-enhanced echocardiography.

**Supplementary Figure 1B.** The anterior leaflet of the MV exhibited prominent SAM (arrow pointed).

**Supplementary Figure 1C.** Severe MR was observed.

**Supplementary Figure 1D.** A high systolic velocity flow in LVOT reached to 5.8m/s with a corresponding peak PG of 136mmHg.

**Supplementary Figure 2A**. The BMD tip was visible on the mid-esophageal long-axis view of TEE (arrow pointed).

**Supplementary Figure 2B.** On the trans-gastric short-axis view, the BMD was shown close to the anterior IVS (arrow pointed).

**Supplementary Figure 2C.** After the hypertrophied myocardium was resected, MR reduced to a mild degree.

**Supplementary Figure 2D.** The systolic peak velocity in the LVOT decreased to less than 3 m/s with a corresponding peak PG of 35mmHg.

**Supplementary Figure 4A.** Upon a seven-month follow-up after surgery, the patient's septal thickness was notably thinner using contrast-enhanced echocardiography.

**Supplementary Figure 4B.** SAM was no longer present (arrow pointed).

**Supplementary Figure 4C.** Moreover, only minimal MR remained.

**Supplementary Figure 4D.** The systolic peak velocity in the LVOT decreased to 2.6 m/s with a corresponding peak PG of 28mmHg.
