# Supplementary material for: Case Report: Mechanical hemolysis resulting from left ventricular outflow tract obstruction after aortic valve replacement relieved by transapical beating-heart septal myectomy
Source: Front Cardiovasc Med. 2024 Jul 11;11:1410222. doi: 10.3389/fcvm.2024.1410222 (PMC11269188; doi:10.3389/fcvm.2024.1410222)
Supplement: Supplementary file 4 [file Datasheet2.pdf]

FIGURE: 1A

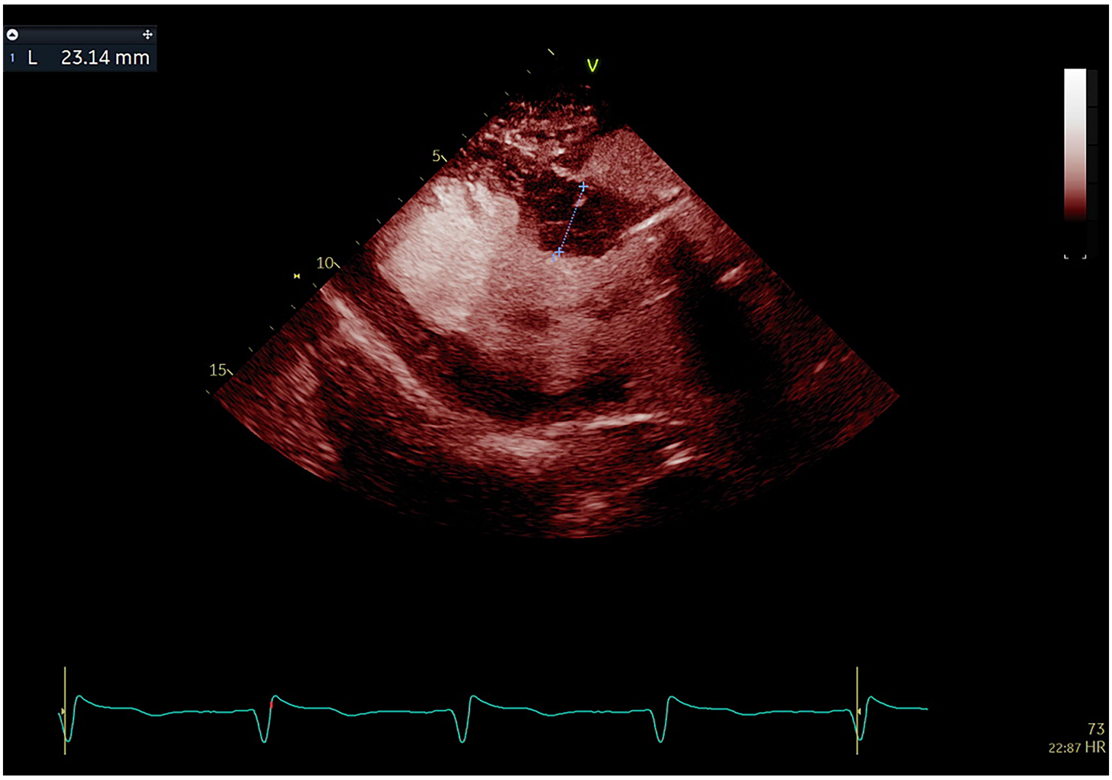

FIGURE: 1B

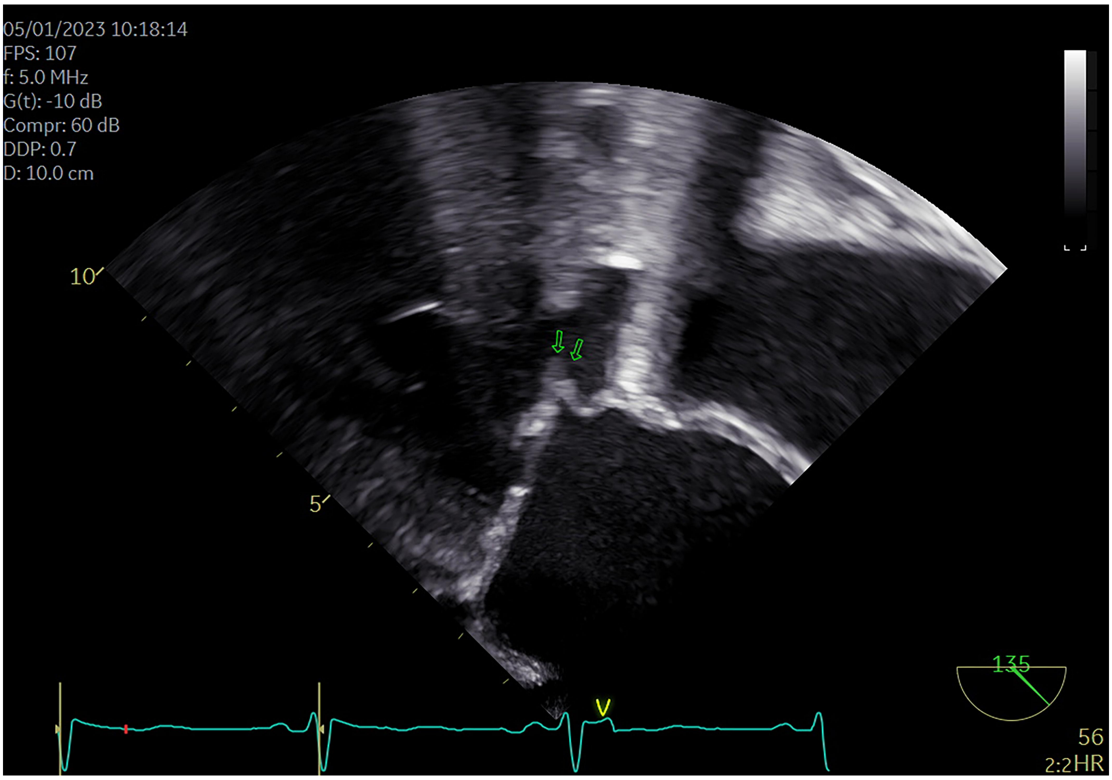

FIGURE: 1C

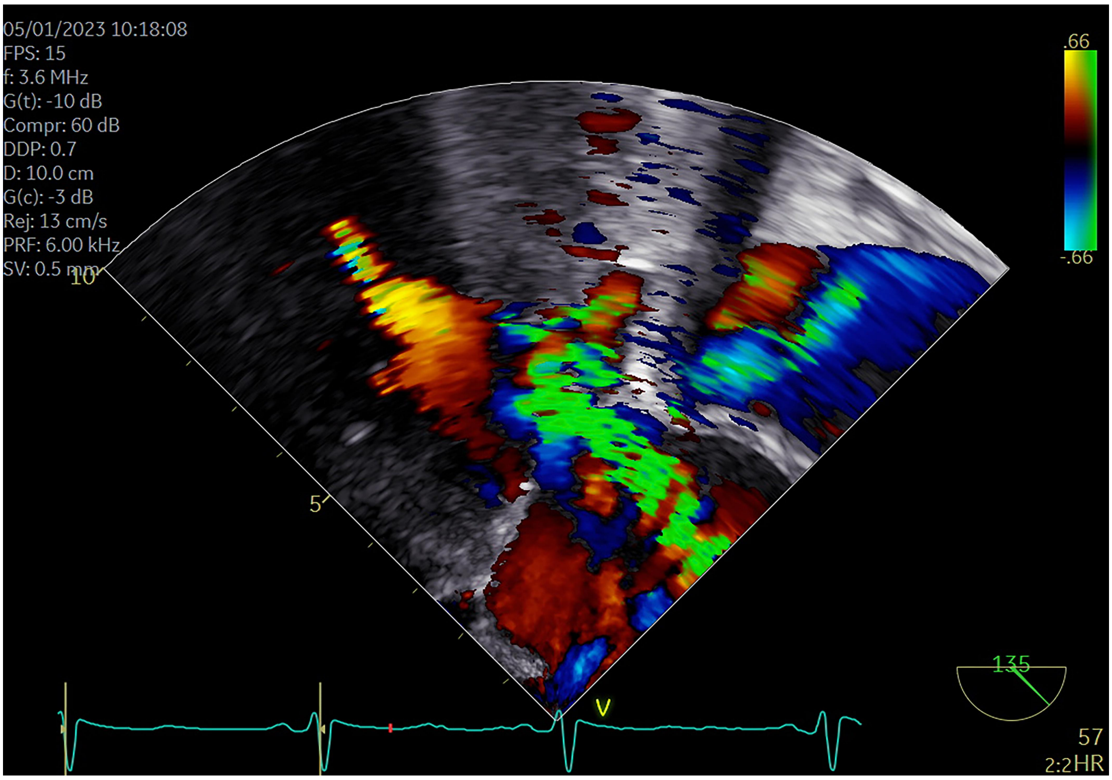

FIGURE: 1D

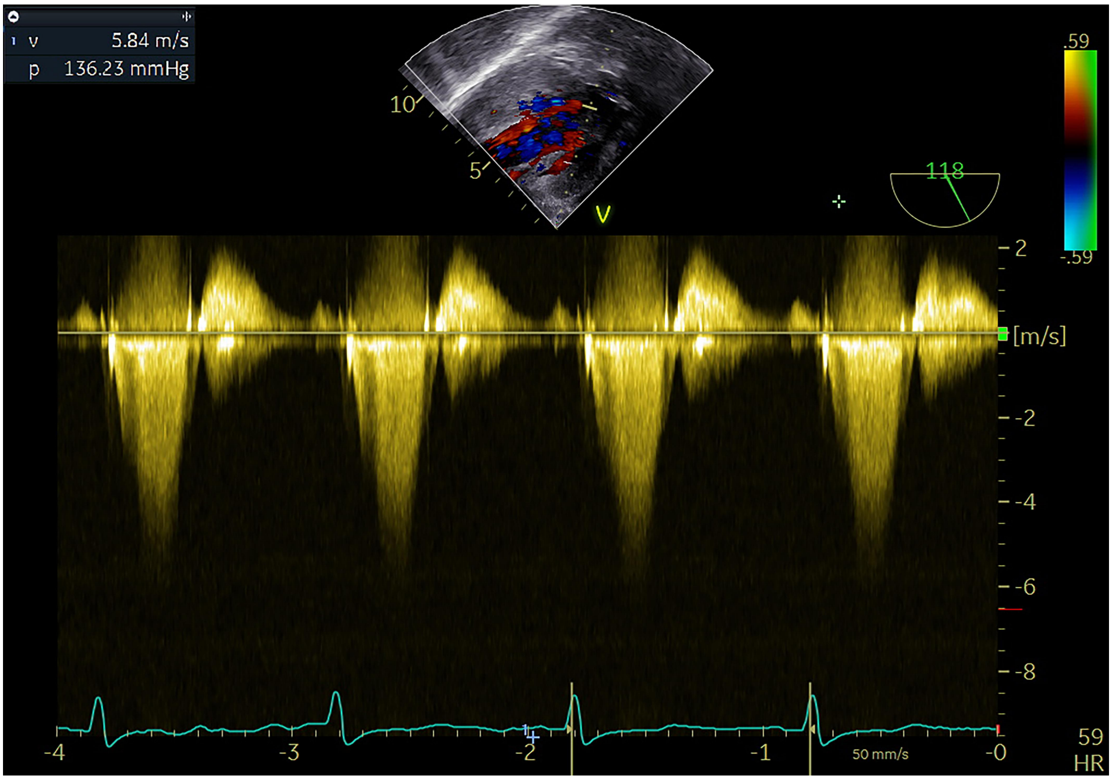

FIGURE: 2A

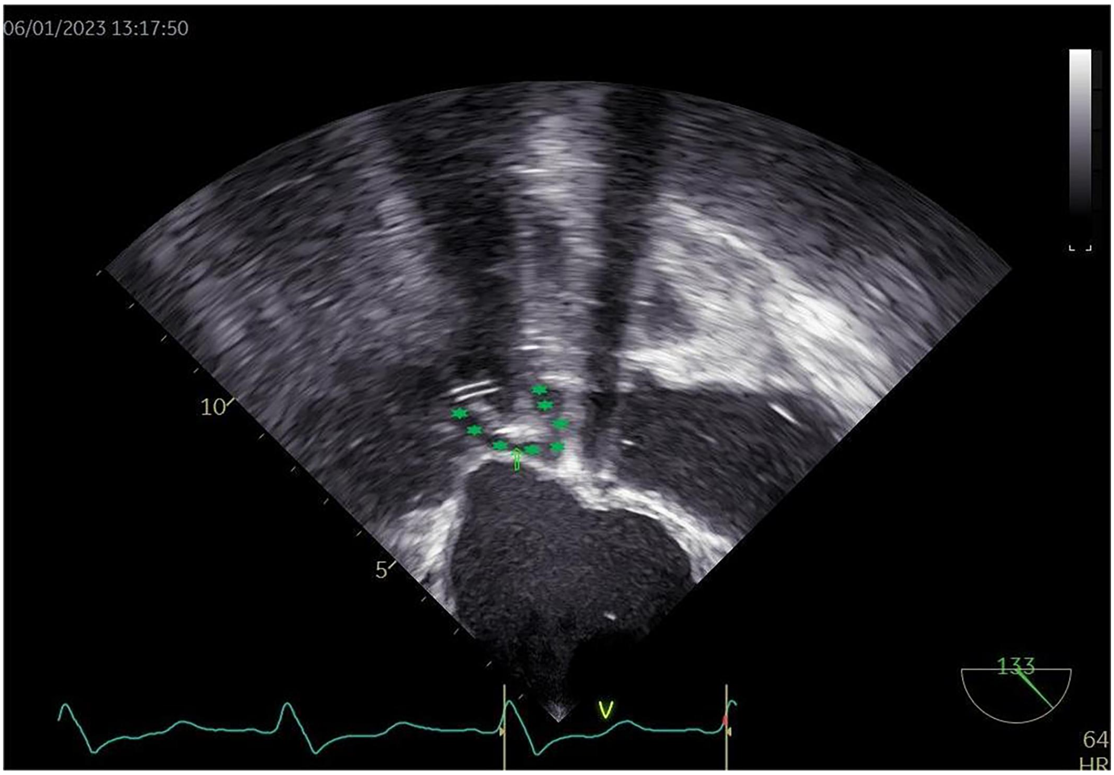

FIGURE: 2B

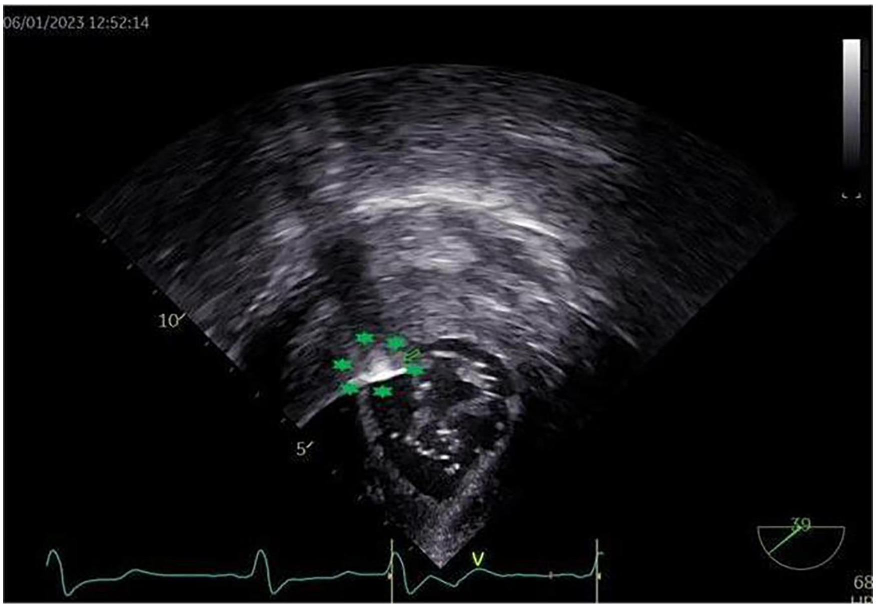

FIGURE:2C

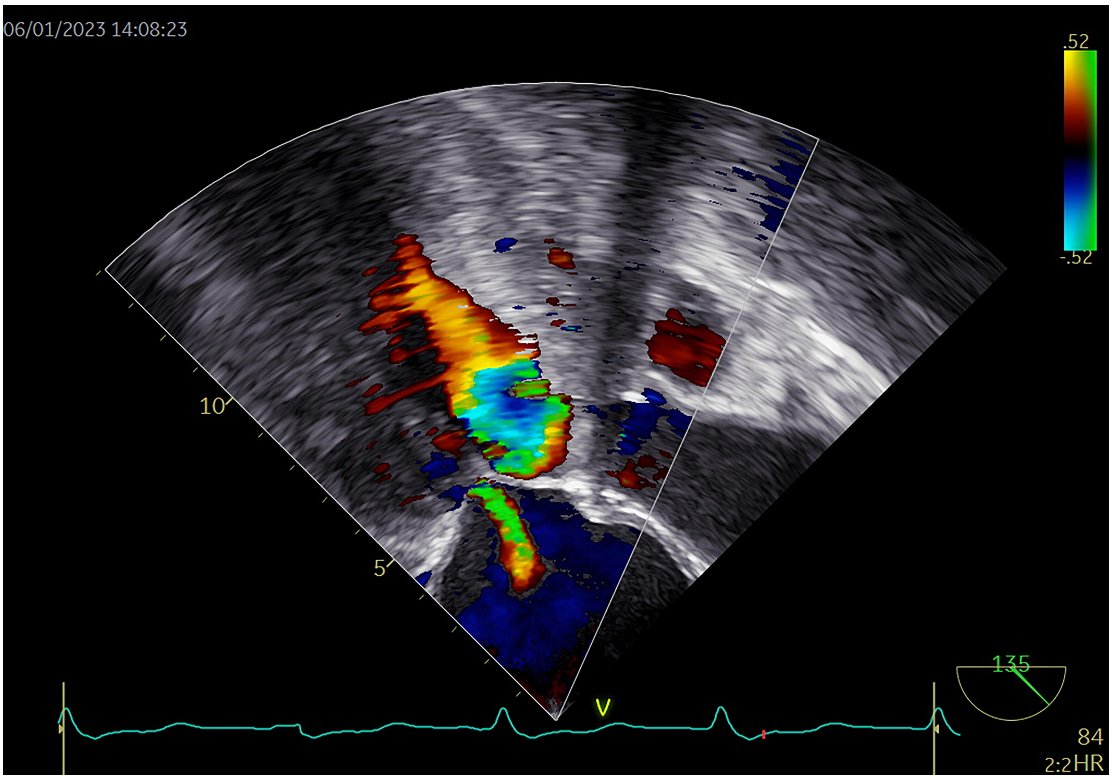

FIGURE: 2D

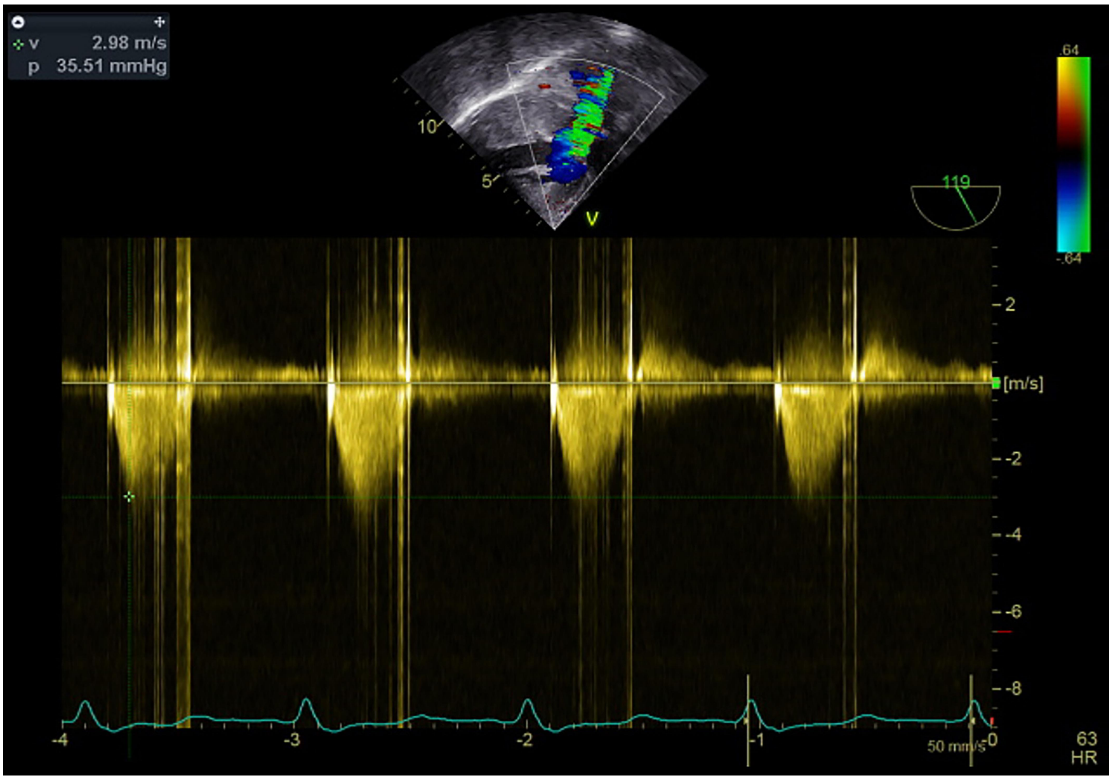

FIGURE: 3

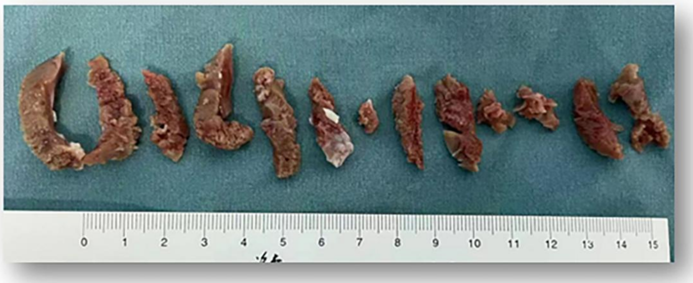

FIGURE: 4A

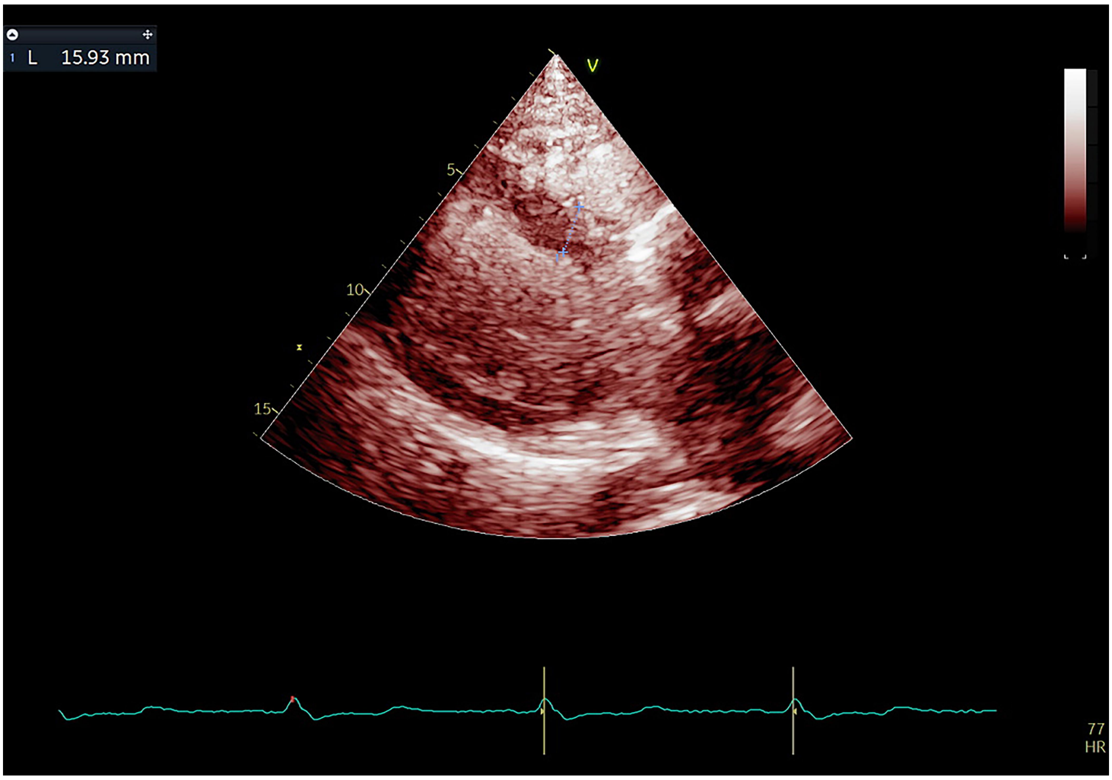

FIGURE: 4B

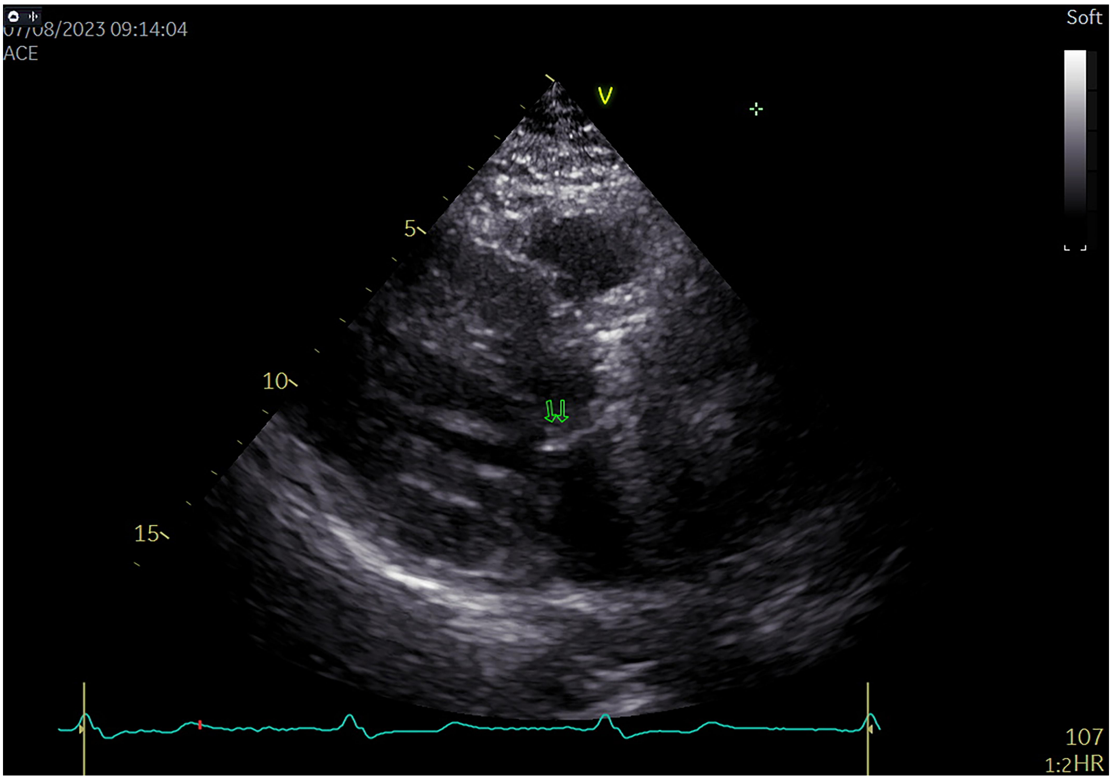

FIGURE: 4C

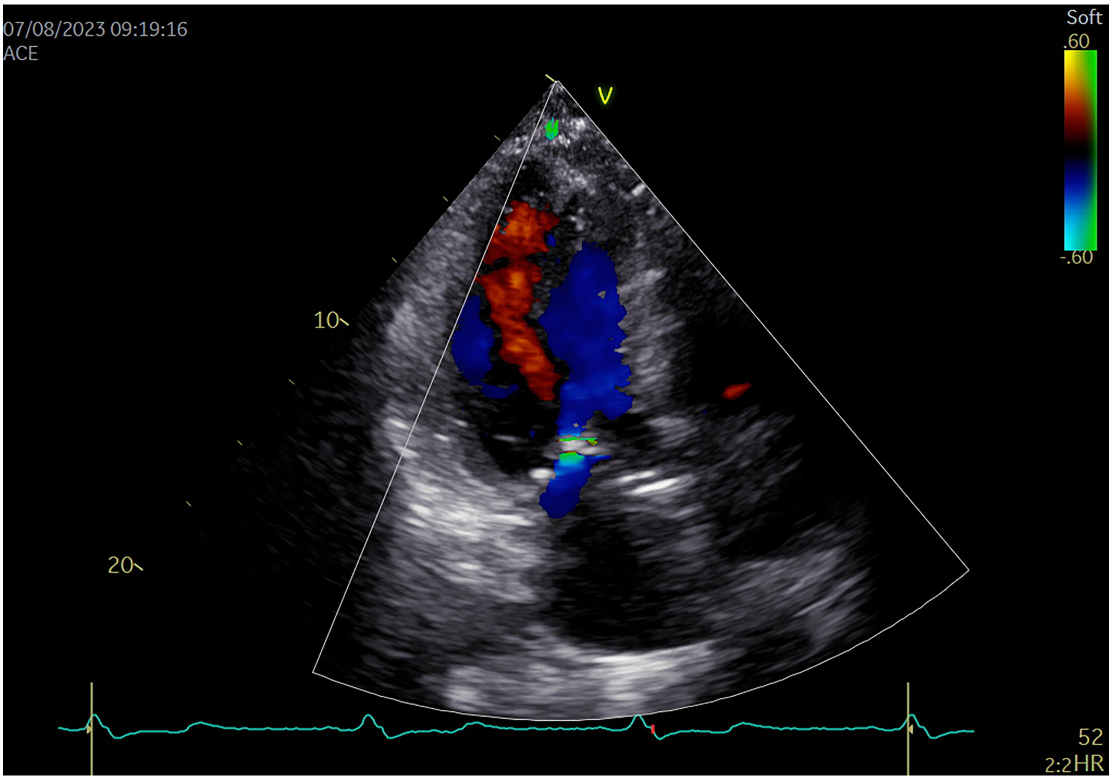

FIGURE: 4D

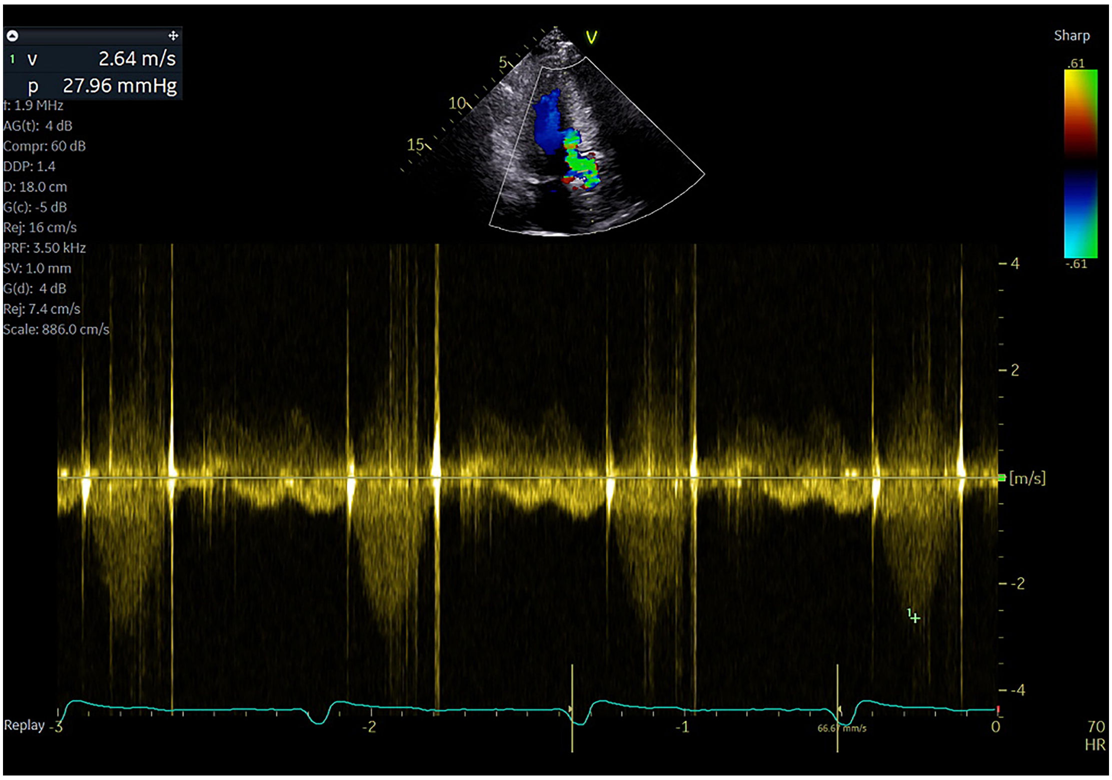
1 v 2.64 m/s
p 27.96 mmHg
f: 1.9 MHz
AG(t): 4 dB
Compr: 60 dB
DDP: 1.4
D: 18.0 cm
G(c): -5 dB
Rej: 16 cm/s
PRF: 3.50 kHz
SV: 1.0 mm
G(d): 4 dB
Rej: 7.4 cm/s
Scale: 886.0 cm/s
Sharp
.61
-.61
V
5
10
15
4
2
[m/s]
-2
-4
Replay
-3
-2
-1
0
70
HR
