# Supplementary figures and images for: Case Report: Mechanical hemolysis resulting from left ventricular outflow tract obstruction after aortic valve replacement relieved by transapical beating-heart septal myectomy
Source: Front Cardiovasc Med. 2024 Jul 11;11:1410222. doi: 10.3389/fcvm.2024.1410222 (PMC11269188; doi:10.3389/fcvm.2024.1410222)

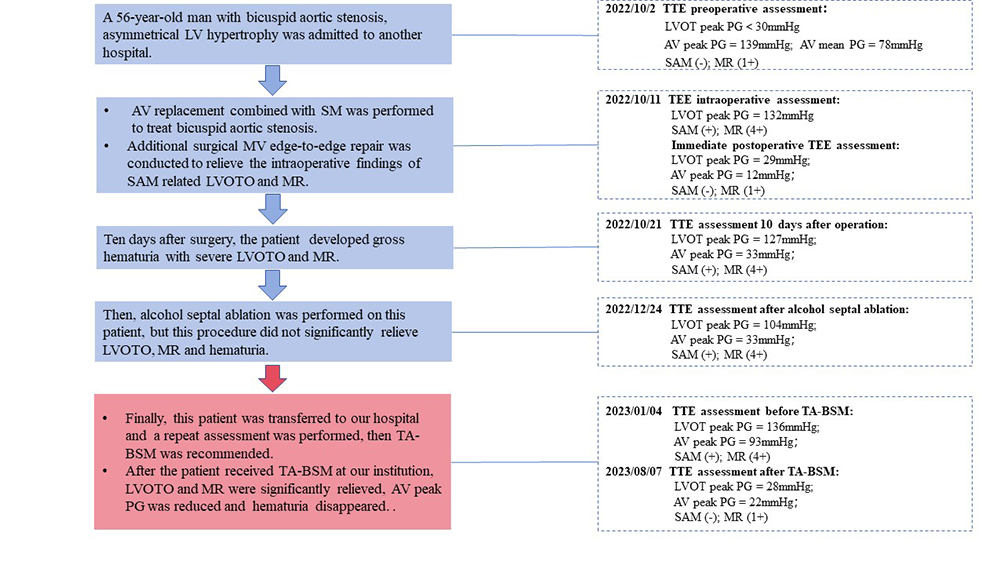

Supplement: Supplementary file 5 [file Image1.tif]
